# Supplementary material for: Interfacial oxygen vacancies yielding long-lived holes in hematite mesocrystal-based photoanodes
Source: Nat Commun. 2019 Oct 23;10:4832. doi: 10.1038/s41467-019-12581-z (PMC6811569; doi:10.1038/s41467-019-12581-z)
Supplement: Supplementary file 1 — Supplementary Info [file 41467_2019_12581_MOESM1_ESM.pdf]

Supplementary Information

Interfacial oxygen vacancies yielding long-lived holes in hematite mesocrystal-based photoanodes

Zhujun Zhang,[1] Izuru Karimata,[1] Hiroki Nagashima,[2] Shunsuke Muto,[3] Koji Ohara,[4] Kunihisa Sugimoto,[1,4,5] and Takashi Tachikawa[1,2]*

[1] Department of Chemistry, Graduate School of Science, Kobe University, 1-1 Rokkodai-cho, Nada-ku, Kobe 657-8501, Japan,

[2] Molecular Photoscience Research Center, Kobe University, 1-1 Rokkodai-cho, Nada-ku, Kobe 657-8501, Japan,

[3] Electron Nanoscopy Section, Advanced Measurement Technology Center, Institute of Materials and Systems for Sustainability, Nagoya University, Furo-cho, Chikusa-ku, Nagoya 464-8603, Japan

[4] Diffraction and Scattering Division, Center for Synchrotron Radiation, Japan Synchrotron Radiation Research Institute, 1-1-1 Kouto, Sayo-cho, Sayo-gun, Hyogo 679-5198, Japan

[5] Institute for Integrated Cell-Material Sciences (iCeMS), Kyoto University, Yoshida-Ushinomiya-cho, Sakyo-ku, Kyoto 606-8501, Japan

* E-mail: tachikawa@port.kobe-u.ac.jp

Supplementary Figures

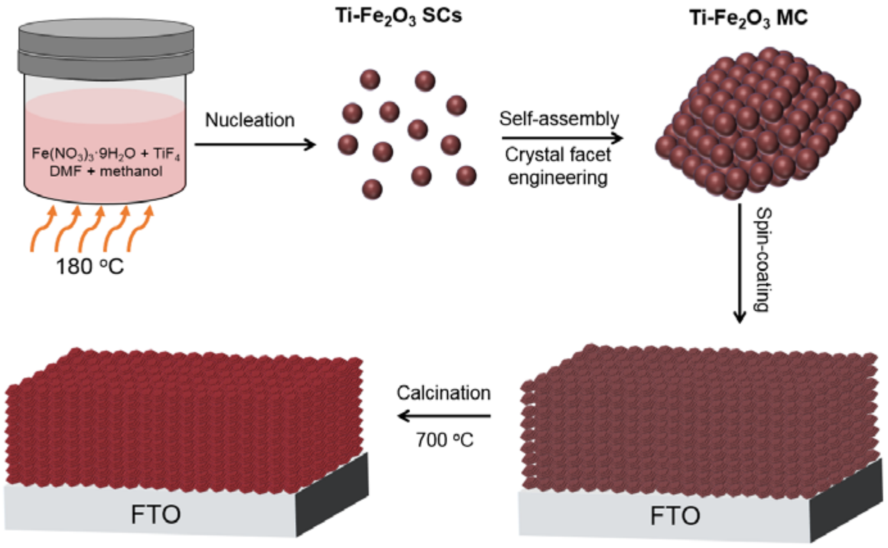

Supplementary Fig. 1 Illustration of the preparation of Ti-Fe_2O_3 MCs and the corresponding photoanode. During solvothermal treatment at 180 °C, the reactants firstly transform to active particles to form the Ti-modified hematite single crystals (Ti-Fe_2O_3 SCs) with sizes of several tens of nanometers, following which the primary nanocrystals self-assemble to MC with an crystallographically aligned orientation by crystal facet engineering.[1] The hematite photoanodes for PEC water oxidation were fabricated via the multiple spin coating of a methanol suspension containing purified Ti-Fe_2O_3 MCs onto a cleaned fluorine-doped tin oxide (FTO) glass. The thickness of the hematite films was controlled by adjusting the number of coating cycles. To increase the contact between MC and FTO glass, as well as to induce interfacial charge transfer, the prepared photoanodes were annealed at 700 °C for 20 min prior to PEC measurements.

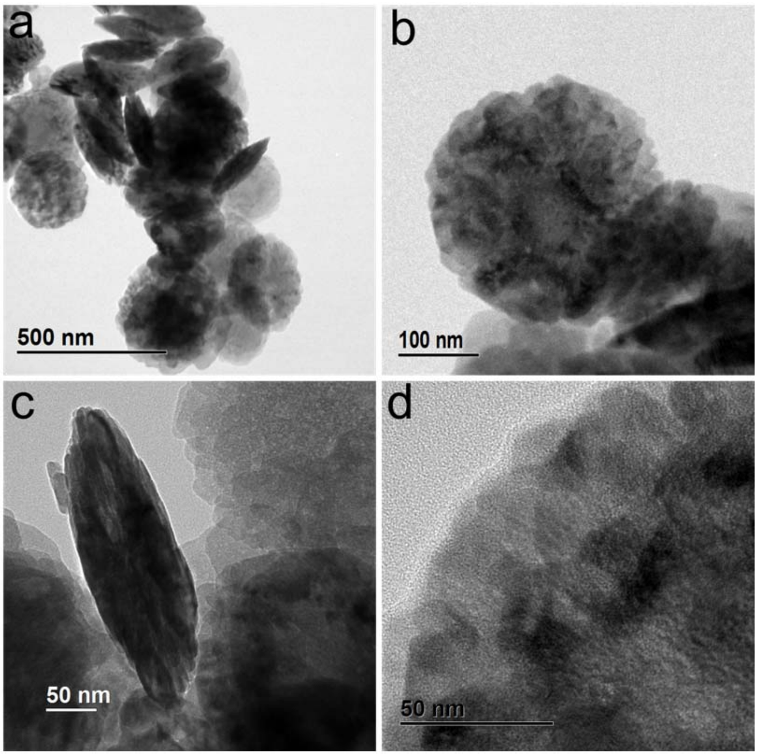

Supplementary Fig. 2 TEM images of as-synthesized Ti-Fe_2O_3 MCs.

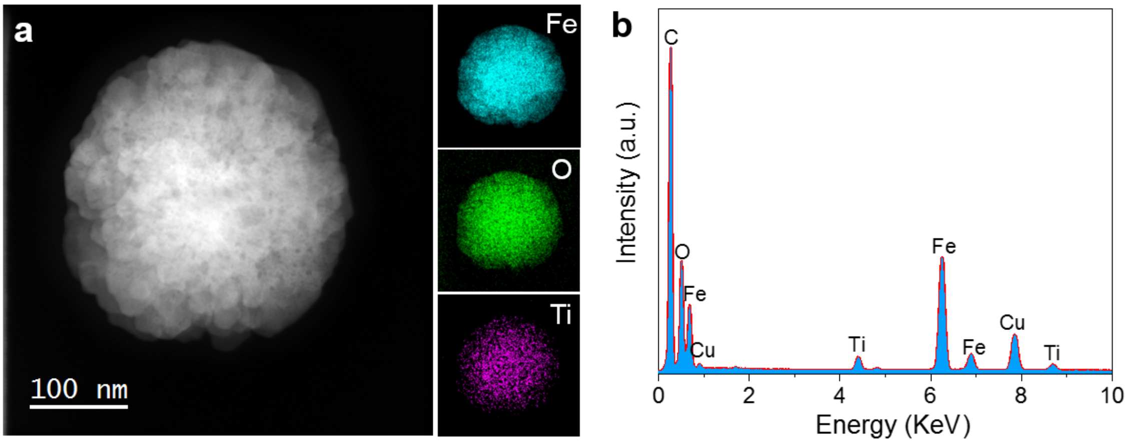

Supplementary Fig. 3 (a) HAADF-STEM (left) and corresponding elemental mapping images (right) of the as-synthesized Ti-Fe_2O_3 MC. (b) EDX spectrum.

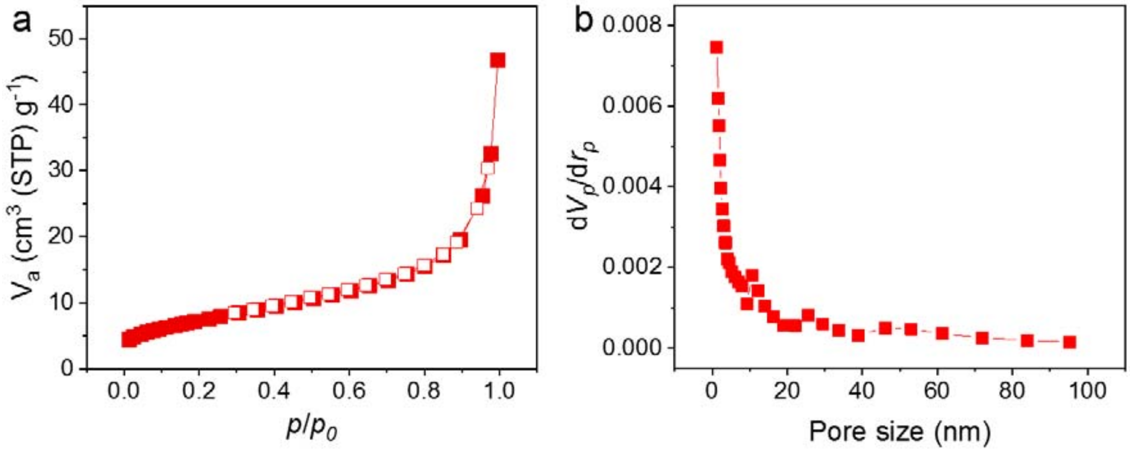

Supplementary Fig. 4 (a) N_2 sorption isotherm and (b) the corresponding pore size distribution curves of as-synthesized Ti-Fe_2O_3 MCs.

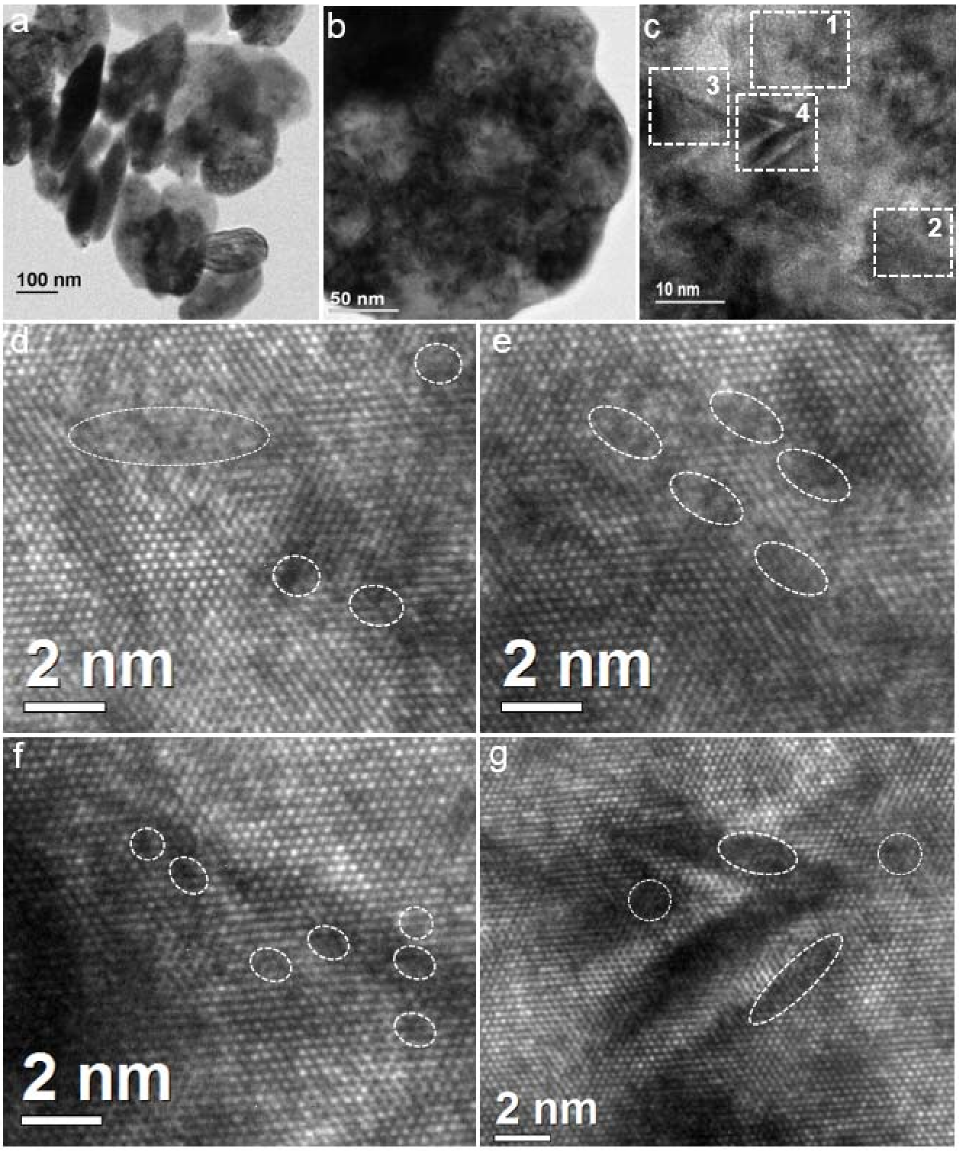

Supplementary Fig. 5 (a-c) TEM images of Ti-Fe_2O_3 MCs collected from the electrode after annealing at 700 °C for 20 min. (d-e) HRTEM images of the selected regions (1-4, respectively) in panel c. The partial sintering of the nanocrystal subunits inside the MC during the annealing, however, induces surface reconstruction at the interfaces (dark and light regions in the images). The regions with sintered interfaces indicate the distorted lattice fringes (e.g., regions surrounded by white dotted lines in panel d-g) suggest the formation of abundant defects at sintered interfaces.[2]

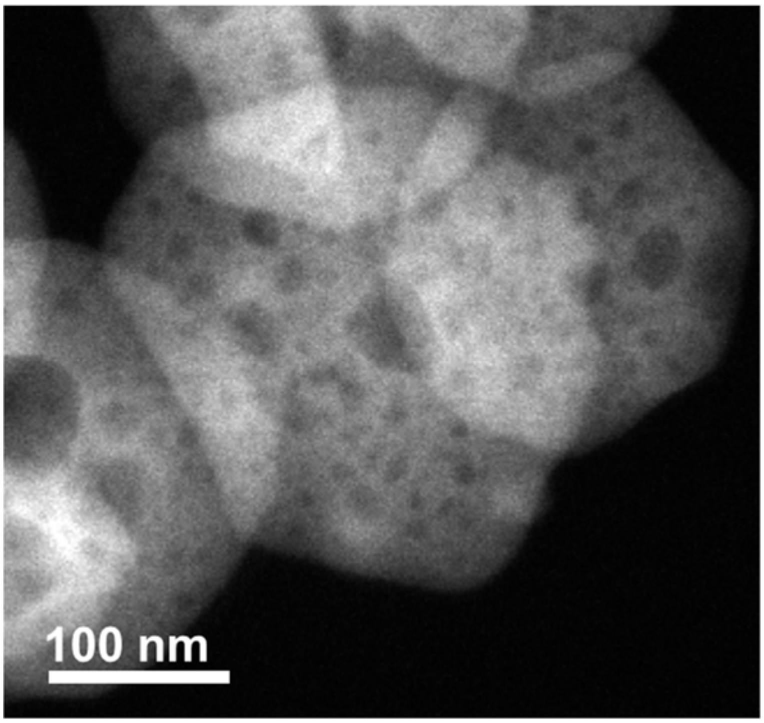

Supplementary Fig. 6 HAADF-STEM image of Ti-Fe_2O_3 MCs collected after annealing at 700 °C for 20 min.

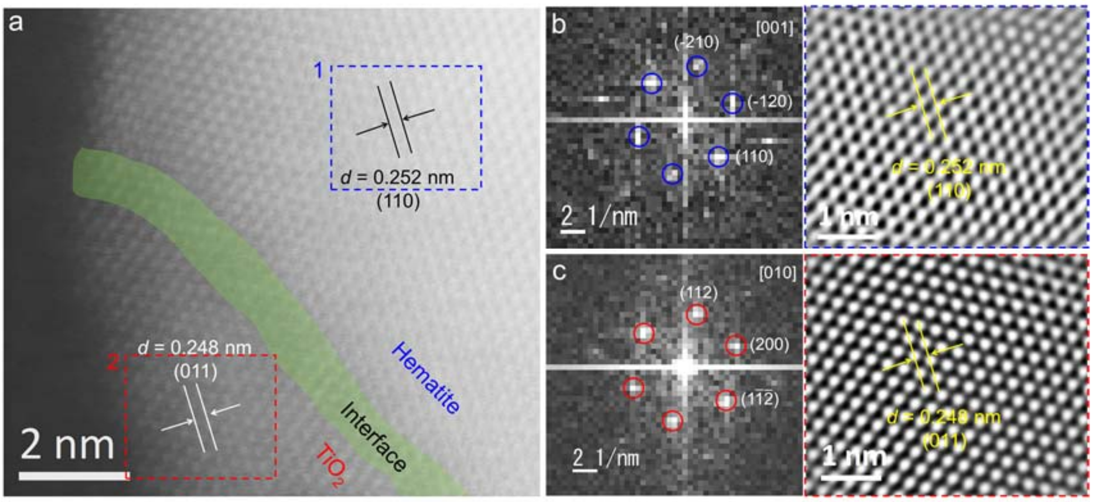

Supplementary Fig. 7 (a) High-resolution HAADF-STEM image obtained near the edge of Ti-Fe_2O_3 MC particle. The corresponding FFT patterns (left) and inverse FFT images (right) of the selected region 1 (b) and region 2 (c) in panel a. The green colored region indicates the interface between hematite and TiO_2.

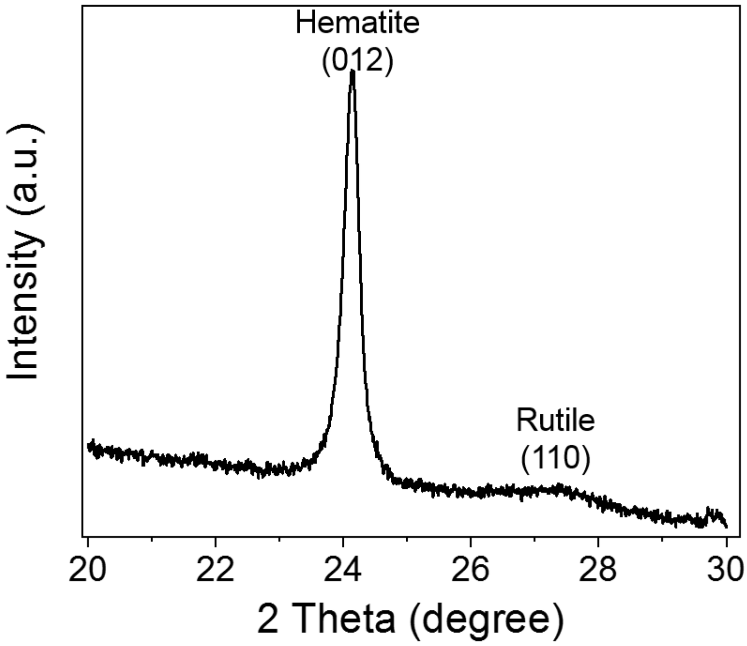

Supplementary Fig. 8 XRD pattern of annealed Ti-Fe_2O_3 MCs measured from 20 to 30 degree with a scanning rate of 0.1 degree min^{-1}.

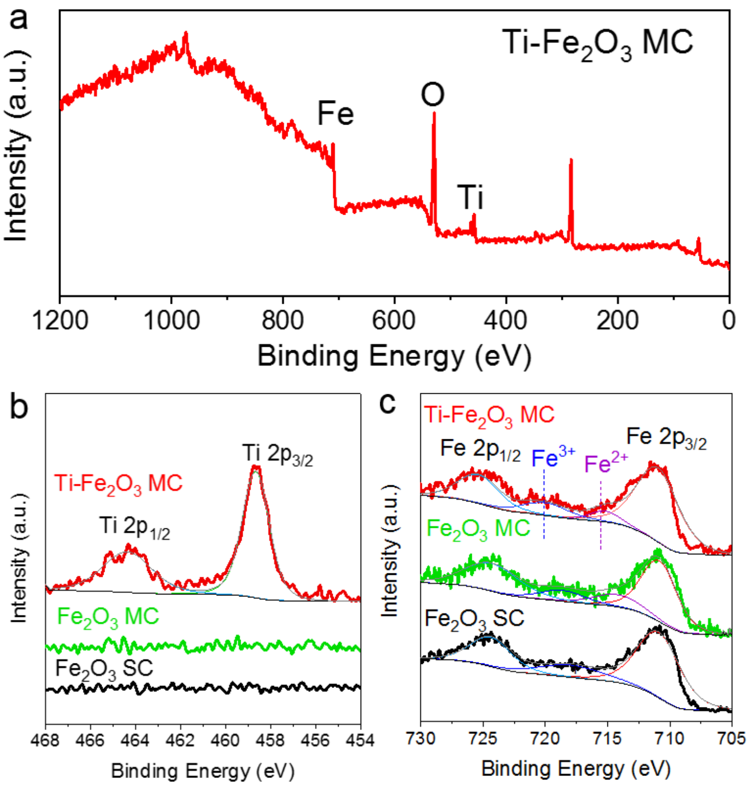

Supplementary Fig. 9 (a) XPS survey spectrum of Ti-Fe_2O_3 MCs, clearly indicating Fe 2p, O 1s, and Ti 2p peaks. (b) XPS Ti 2p and (c) Fe 2p spectra of Ti-Fe_2O_3 MCs, Fe_2O_3 MCs, and Fe_2O_3 SCs. In panel b, only the Ti-Fe_2O_3 MC sample exhibited the characteristic peaks at binding energies of 458.6 (Ti $2p_{1/2}$) and 464.3 eV (Ti $2p_{3/2}$). In panel b, the Fe 2p XPS spectra of the samples represent the doublet Fe $2p_{3/2}$ and Fe $2p_{1/2}$ signals of hematite with binding energies of 710.8 and 724 eV, respectively. A satellite band at approximately 719 eV was observed for all the samples, indicating the presence of Fe^{3+} species.[3] Additionally, another weak satellite band at approximately 716 eV was observed for MCs, which can be assigned to Fe^{2+} species.[3]

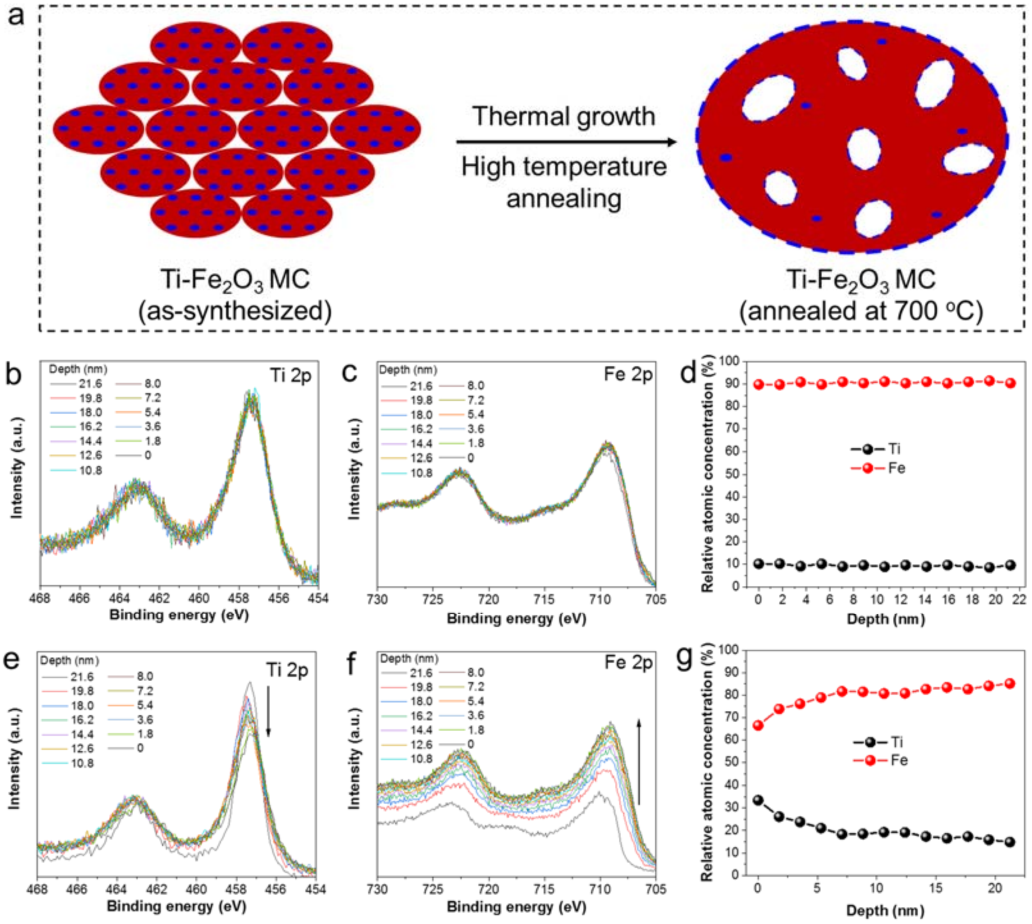

Supplementary Fig. 10 (a) Scheme illustration of thermal growth of TiO_2 on hematite MCs. XPS depth profiles of Ti 2p (b) and Fe 2p (c) for as-synthesized Ti-Fe_2O_3 MCs. (d) The relative atomic concentration of Ti and Fe for different depth of as-synthesized Ti-Fe_2O_3 MCs. XPS depth profiles of Ti 2p (e) and Fe 2p (f) for Ti-Fe_2O_3 MCs annealed at 700 °C. (g) The relative atomic concentration of Ti and Fe for different depth of Ti-Fe_2O_3 MCs annealed at 700 °C. Since XPS mainly explores the surface of the material, the depth profiles were examined to analyze the compositions inside the MC. The results clearly indicate that the percentage of Ti (Fe) ions decreased (increased) by etching to approximately 20 nm depth for annealed Ti-Fe_2O_3 MCs, while those remained unchanged for the unannealed sample. The abundant presence of Ti species imply that Ti ions were mostly segregated from the bulk to the outer surfaces.

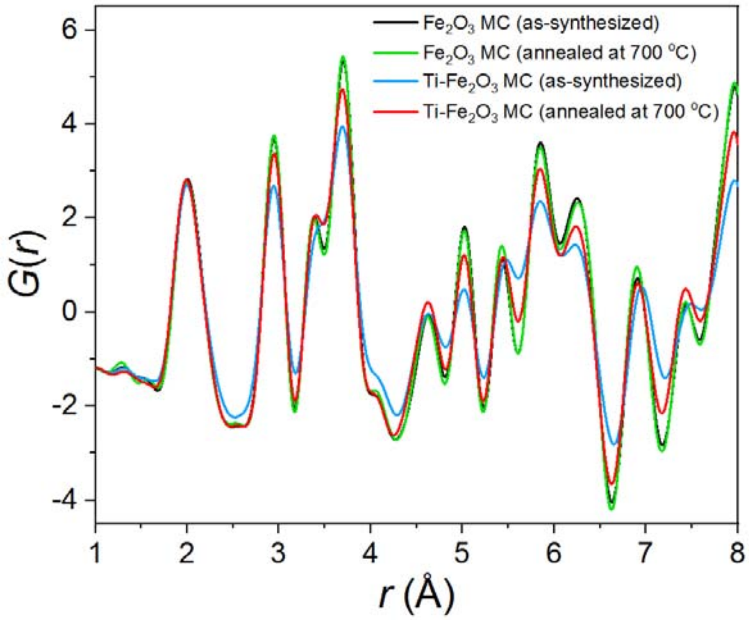

Supplementary Fig. 11 The synchrotron-based X-ray total scattering and pair distribution function (PDF) analyses of the samples. The peak at around 2 Å is composed of short Fe−O (1.94 Å) and long Fe−O (2.12 Å) distances.[4] The peak at around 3 Å is composed of the first neighbor edge-sharing (2.97 Å) and face-sharing (2.89 Å) Fe−Fe distances. The first neighbor corner-sharing Fe−Fe pairs show two peaks at 3.39 Å and 3.70 Å.[5] After the annealing of as-synthesized Fe_2O_3 MCs at 700 °C, there was no apparent change in the peaks corresponding to Fe−O and Fe−Fe distances, possibly due to the low concentration of oxygen vacancies in MCs (see black and green lines). In contrast, a significant difference in the peaks was observed for Ti-modified Fe_2O_3 MCs before and after the annealing (see blue and red lines). By the annealing of as-synthesized Ti-Fe_2O_3 MCs, all the peaks became narrow and similar to those of pure hematite, suggesting that most of doped Ti ions are segmented from the bulk. Notably, the difference in the peak intensities between annealed Fe_2O_3 (green line) and Ti-Fe_2O_3 (red line) MCs implies that a small portion of Ti ions are doped in hematite. For Fe_2O_3 MCs, no apparent difference in Fe–O and Fe–Fe bond distances was observed between the samples before and after the annealing. This is probably due to the fact that the amounts of V_O in annealed MCs are still too small to be detected. Whereas, the Ti modification results in a significant intensity decrease and broadening of the peaks. After the annealing, the spectral features are almost restored to those of pure hematite, indicating that most Ti ions are segmented from the bulk to the surface while leaving a trace as dopant.

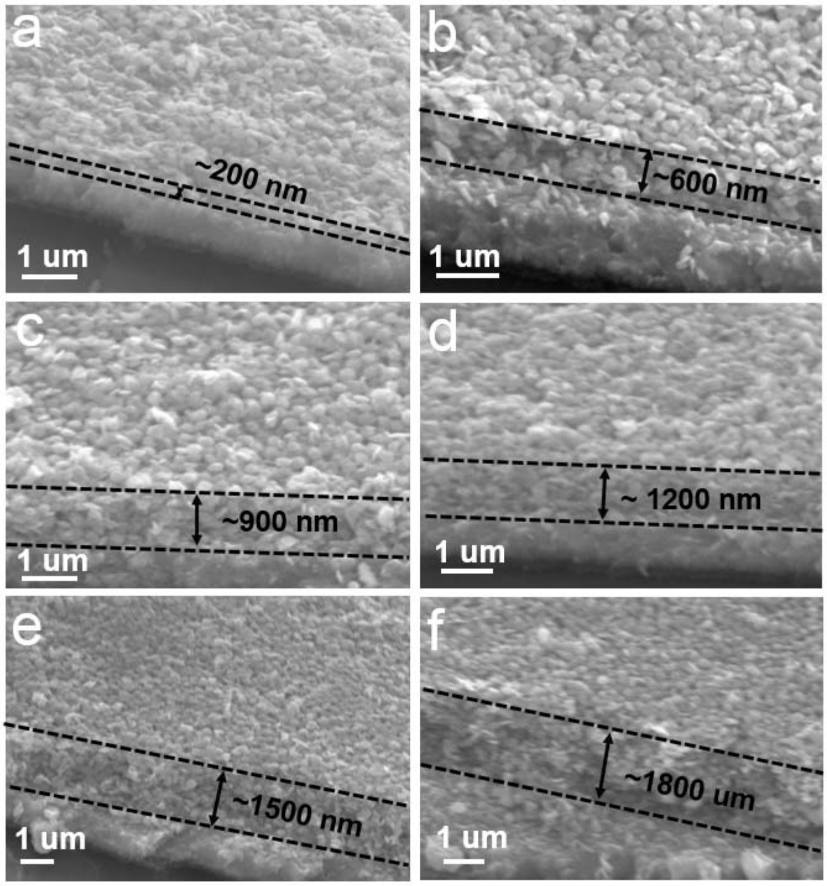

Supplementary Fig. 12 Cross-sectional SEM images of Ti-Fe_2O_3 MC photoanodes prepared by different spin-coating cycles: (a) 10 cycles; (b) 20 cycles; (c) 30 cycles; (d) 40 cycles; (e) 50 cycles; (f) 60 cycles. The film thickness can be controlled ranging from ~200 to ~1800 nm by adjusting the number of spin coating cycles from 10 to 60.

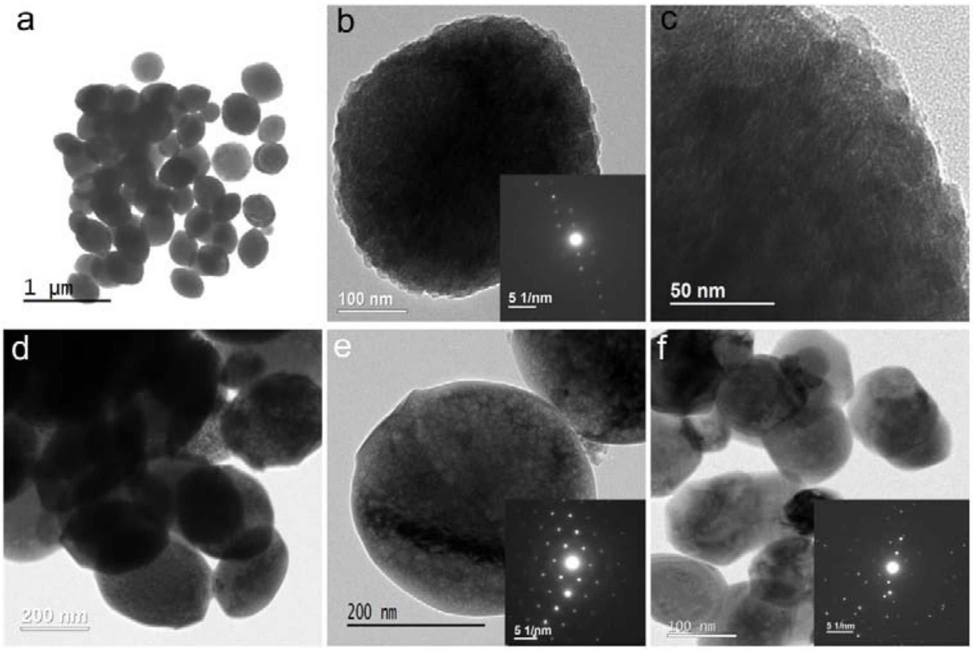

Supplementary Fig. 13 TEM images of as-synthesized Fe_2O_3 MCs (a, b, and c), Fe_2O_3 MCs collected after annealing at 700 ^oC for 20 min (d and e), and Fe_2O_3 SCs collected after annealing at 700 ^oC for 20 min (f). Inset: the corresponding SAED images.

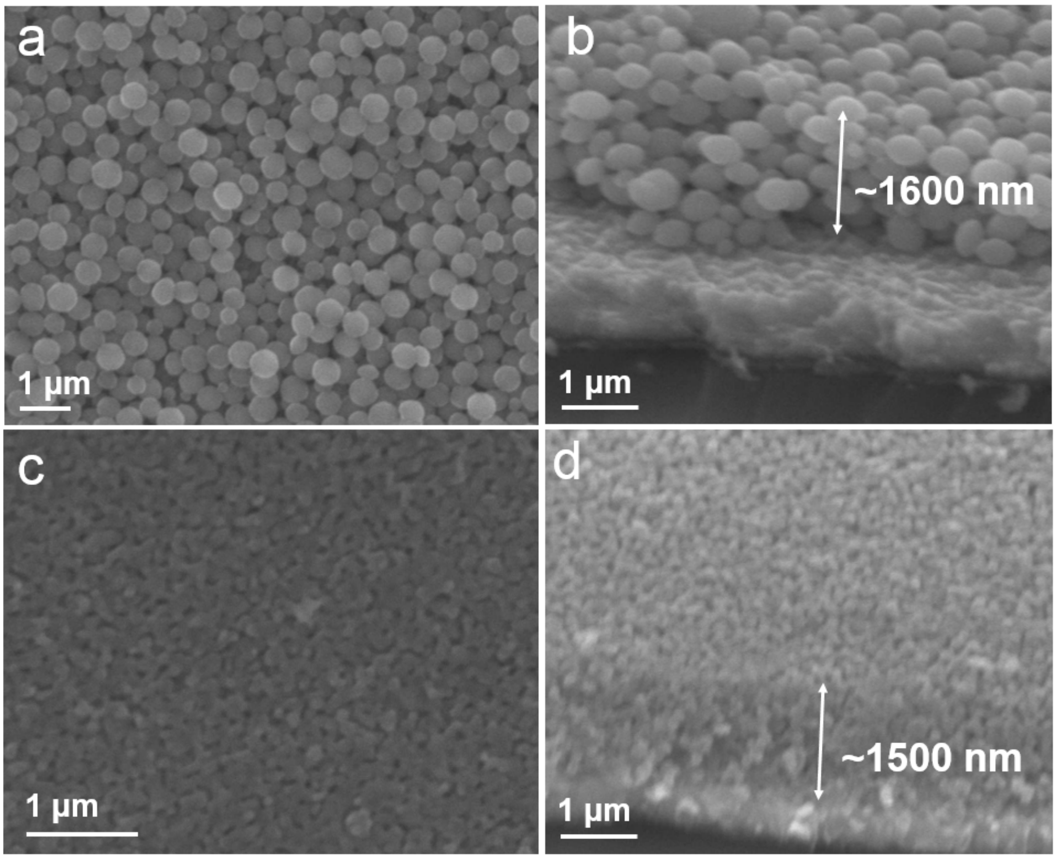

Supplementary Fig. 14 (a) Top-view and (b) cross-sectional SEM images of Fe_2O_3 MC photoanode. (c) Top-view and (d) cross-sectional SEM images of Fe_2O_3 SC photoanode.

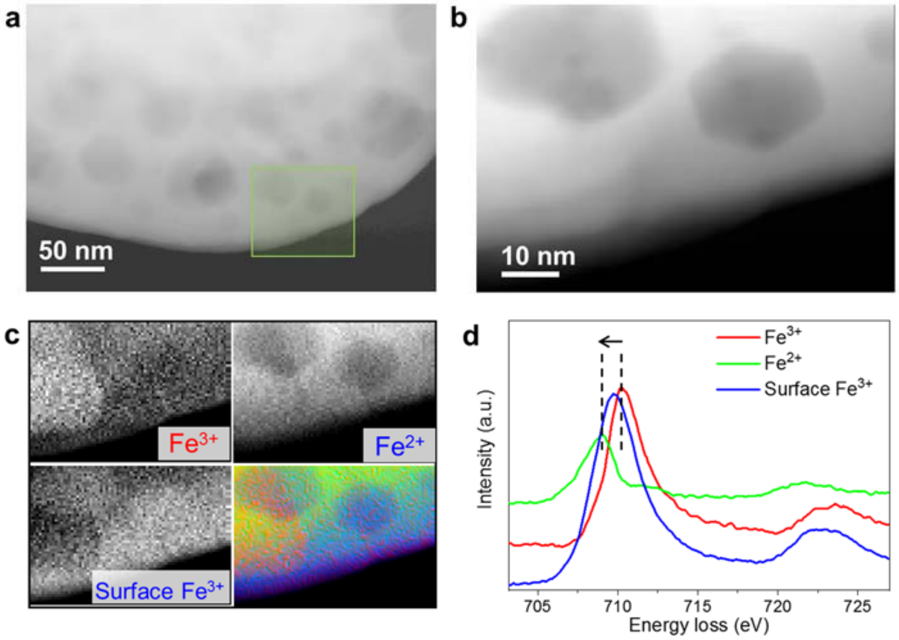

Supplementary Fig. 15 (a,b) HAADF-STEM image of Fe_2O_3 MC. (c) Corresponding EELS chemical composition maps from the region of panel b. The merged image of component 1 (red), 2 (green), and 3 (blue) is also shown. (d) The spectra of corresponding components were isolated by multivariate analysis.

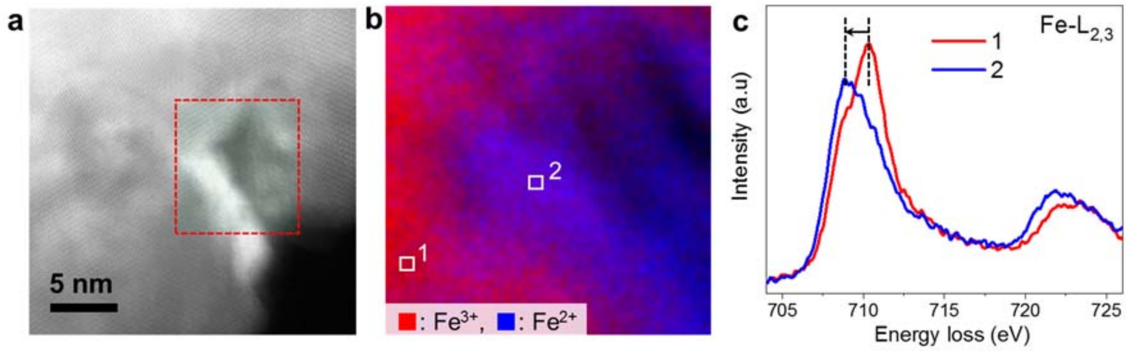

Supplementary Fig. 16 (a) High-resolution HAADF-STEM image. (b) The EELS Fe-$L_{2,3}$ map of the selected region in panel a. This image was obtained using multiple linear least-square fit to the spectrum image data with the reference spectra extracted from the well-defined areas in panel a. (c) Fe-$L_{2,3}$ spectra of the selected regions in panel b. The valence states of iron in Ti-Fe_2O_3 MC were further identified by Fe-$L_{2,3}$ EEL spectra and separately visualized. Fe^{2+} ions (i.e., V_O)[6] are mostly distributed in the bright zone where the distorted lattice fringes are seen, while Fe^{3+} ions are distributed in the dark zone where clear lattice fringes are seen.

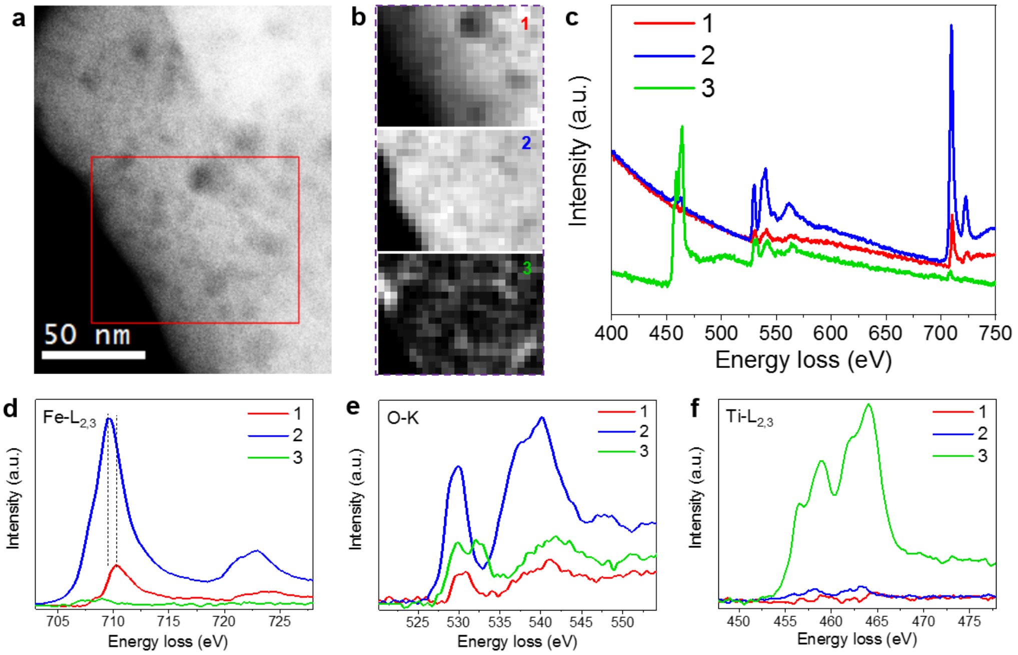

Supplementary Fig. 17 (a) HAADF-STEM image of Ti-Fe_2O_3 MC. STEM-EELS spectrum image data was obtained from the framed area inset. (b) Corresponding EELS chemical composition maps. (c) Corresponding component spectra isolated by multivariate curve resolution analysis. EEL spectra of Fe $L_{2,3}$ (d), O K (e), and Ti $L_{2,3}$ (f). The component 1 is considered to be mainly attributed to hematite according to the similarity of Fe-$L_{2,3}$ and O-K spectra with the reference.[7] For component 2, the shift of Fe-$L_{2,3}$ peaks to lower energy loss and the suppression of the pre-peak intensity at ca. 527 eV of O-K spectrum indicate the presence of Fe^{2+} species. These changes can be explained by the formation of 4-coordinated Fe^{2+}.[8,9] Here, we propose that their possible origin is not only the oxygen vacancies at the interfaces in MCs, but also $Fe_{2-x}Ti_xO_3$ (e.g., ilmenite ($FeTiO_3$)), judging from the Ti-$L_{2,3}$ spectral profile.[10] The latter is considered to be present at the interface between hematite and TiO_2 (see Supplementary Fig. 7) and play a key role in the formation of the TiO_2 overlayer during the annealing at 700 °C. The component 3 is mainly TiO_2. Considering the similarity of Ti-$L_{2,3}$ and O-K spectra with the reference,[10,11] the typical transition temperature from anatase to rutile at ca. 600 °C,[12] and XRD data (see Supplementary Fig. 8), rutile phase would be a potential candidate. The formation of rutile TiO_2 on hematite has been reported in the literature.[4] It should be noted that component 3 contains a small amount of Fe species. This result also supports the presence of the $Fe_{2-x}Ti_xO_3$ phases.

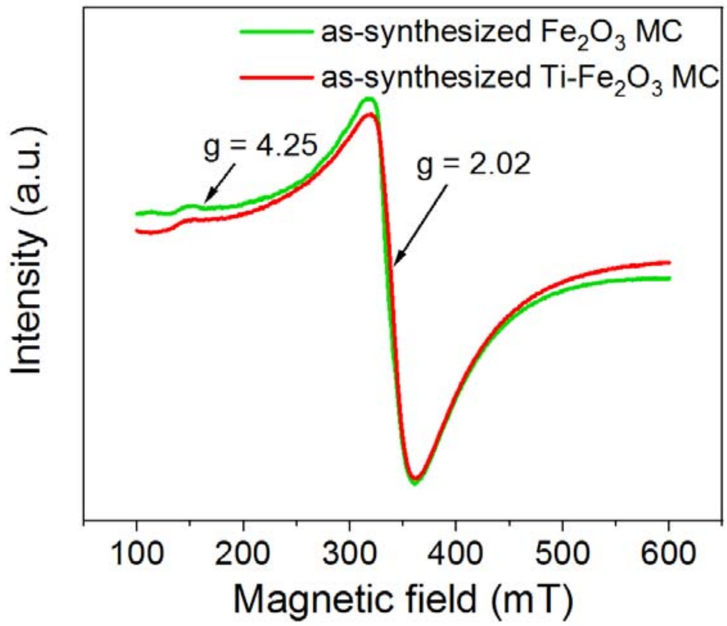

Supplementary Fig. 18 EPR spectra of as-synthesized Ti-Fe_2O_3 MCs and Fe_2O_3 MCs.

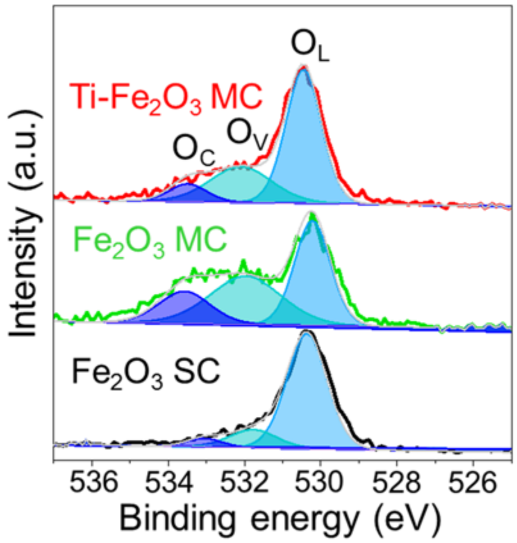

Supplementary Fig. 19 O 1s XPS of the samples. Because the formation of V_O alters the coordination of O atoms and chemical valence state of Fe cations in hematite,[13-16] the oxygen species at the surface and exposed interfaces were characterized by the O 1s XPS spectra. Peaks with binding energies at approximately 530.2, 531.9, and 533.3 eV were observed for the samples, corresponding to the O^{2-} species in the hematite lattice (O_L), hydroxyl groups (OH^-) bonded to the metal cations (Fe−OH) in the oxygen deficient region (O_V), and chemisorbed or dissociated oxygen species from the H_2O molecules (O_C), respectively.[13] In particular, the contents of O_V in both Ti-Fe_2O_3 and Fe_2O_3 MCs are much higher than that in Fe_2O_3 SCs, indicating the formation of abundant Vo in the MCs.

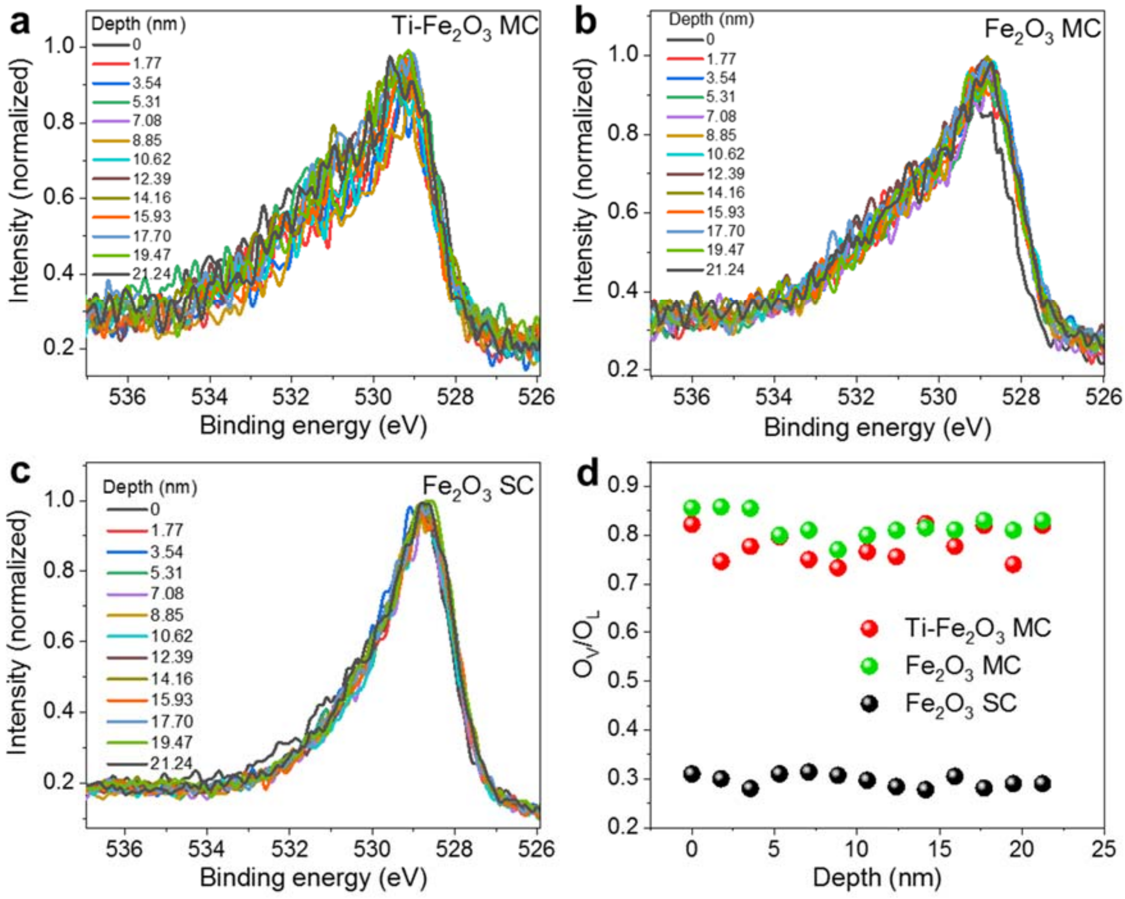

Supplementary Fig. 20 O 1s XPS depth profiles of Ti-Fe_2O_3 MC (a), Fe_2O_3 MC (b), and Fe_2O_3 SC (c) samples. (d) The relative O_V/O_L area ratios at different depths. It is clear that the concentration of V_O remains unchanged from the surface to the bulk region (at approximately 20 nm depth) for both Ti-Fe_2O_3 MC and Fe_2O_3 MC samples.

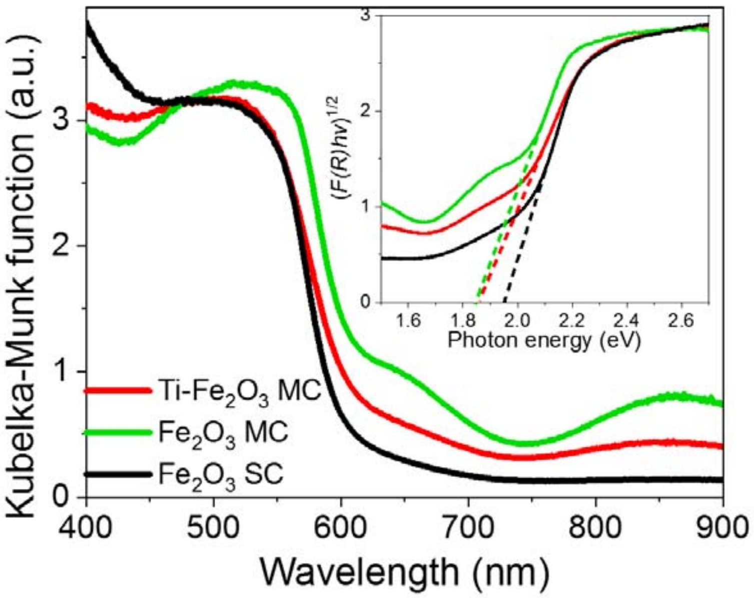

Supplementary Fig. 21 Steady-state UV-Visible diffuse reflectance spectra as Kubelka-Munk function of the samples. Inset: The corresponding plot of $(F(R)h\nu)^{1/2}$ versus $h\nu$. The MC samples possess significant absorption in the longer wavelength region (>650 nm), which was attributed to the Urbach tail and defect states, such as V_O in hematite.[17] The band gaps of Ti-Fe_2O_3 and Fe_2O_3 MC photoanodes were measured to be approximately 1.85 eV, which is ~0.1 eV smaller than that of the Fe_2O_3 SC photoanode (1.95 eV). The slightly narrower band gap of Ti-Fe_2O_3 and Fe_2O_3 MC samples was partly due to the V_O overlapped with the valence band edge, thus leading to the raise of valence band position.[18]

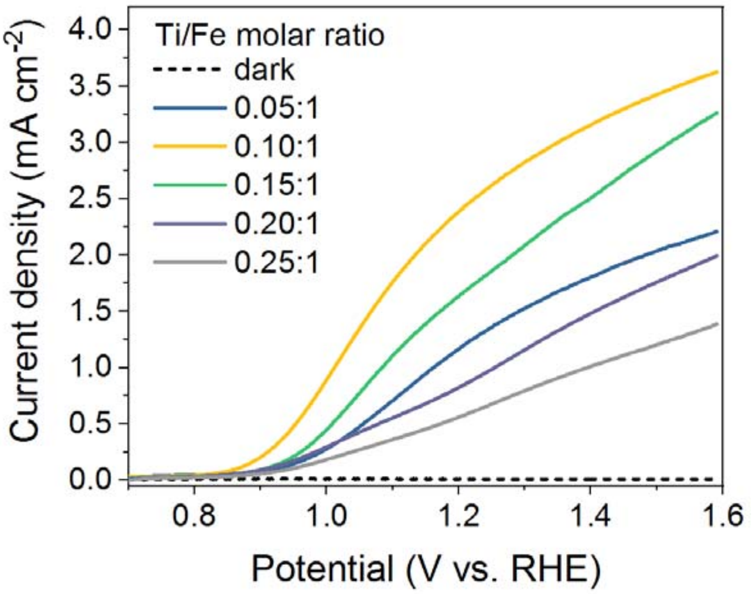

Supplementary Fig. 22 The current density-voltage curves of Ti-Fe_2O_3 MC photoanodes (50 cycles of spin-coating) prepared at different Ti/Fe molar ratios under back illumination with AM 1.5 G simulated sunlight in 1.0 M NaOH.

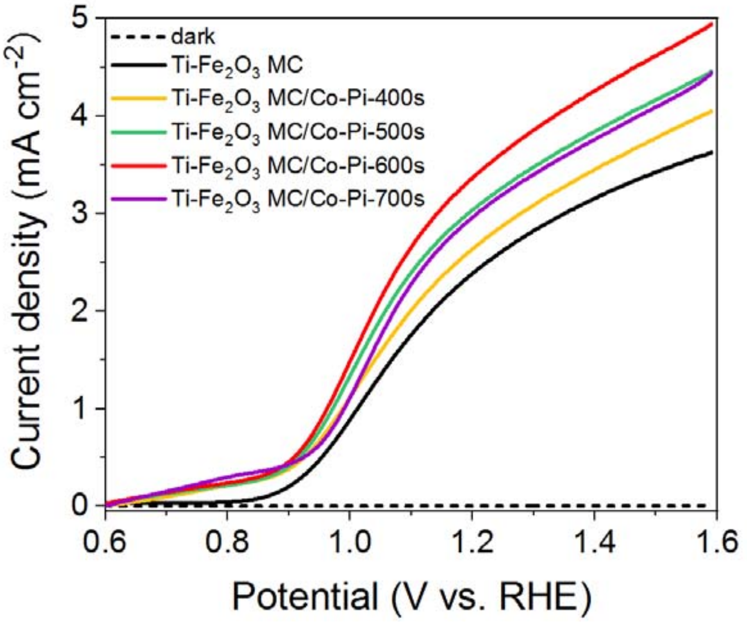

Supplementary Fig. 23 The current density-voltage curves of Co-Pi-modified Ti-Fe_2O_3 MC photoanodes prepared by photo-assisted electrodeposition at different deposition times.

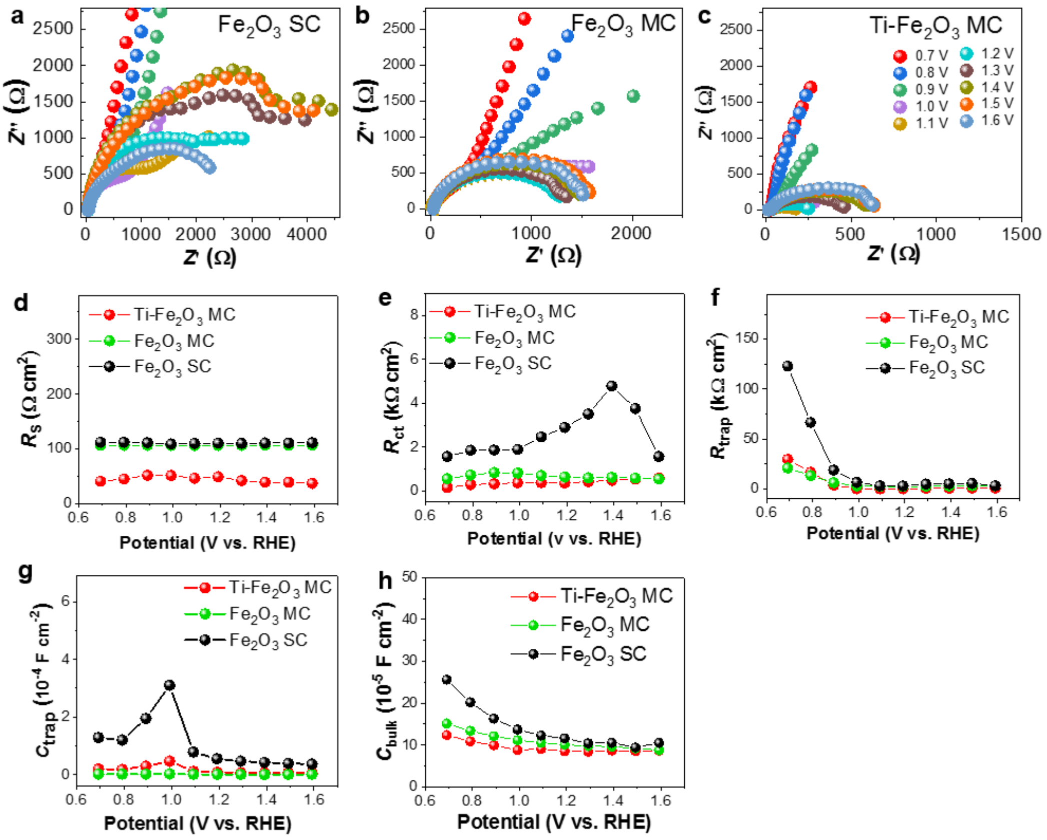

Supplementary Fig. 24 Photoelectrochemical impedance spectroscopy (PEIS) for (a) Fe_2O_3 SC; (b) Fe_2O_3 MC, and (c) Ti-Fe_2O_3 MC photoanodes measured at different applied potentials under back illumination. The fitted results for R_S (d), R_{ct} (e), R_{trap} (f), C_{trap} (g), and C_{bulk} (h).

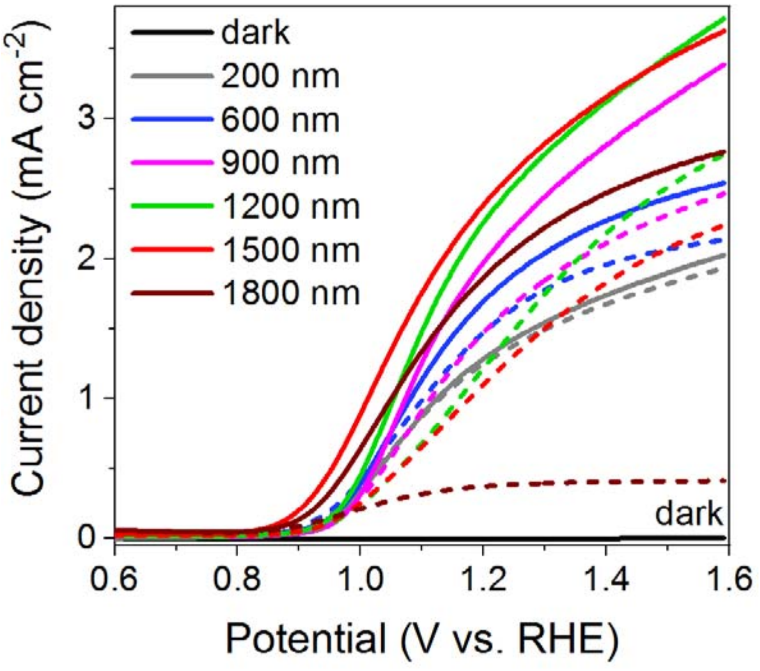

Supplementary Fig. 25 The current density-voltage curves of Ti-Fe_2O_3 MC photoanodes with different film thickness via back illumination (solid lines) and front illumination (dash lines) with AM 1.5 G simulated sunlight in 1.0 M NaOH.

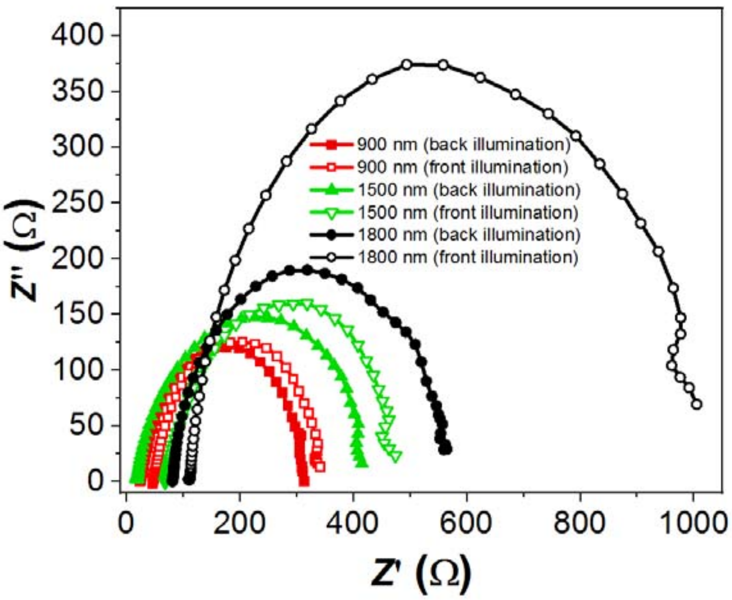

Supplementary Fig. 26 Electrochemical impedance spectra measured for the Ti-Fe_2O_3 MC samples with different thickness at 1.23 V vs. RHE under back illumination or front illumination with AM 1.5 G simulated sunlight in 1.0 M NaOH.

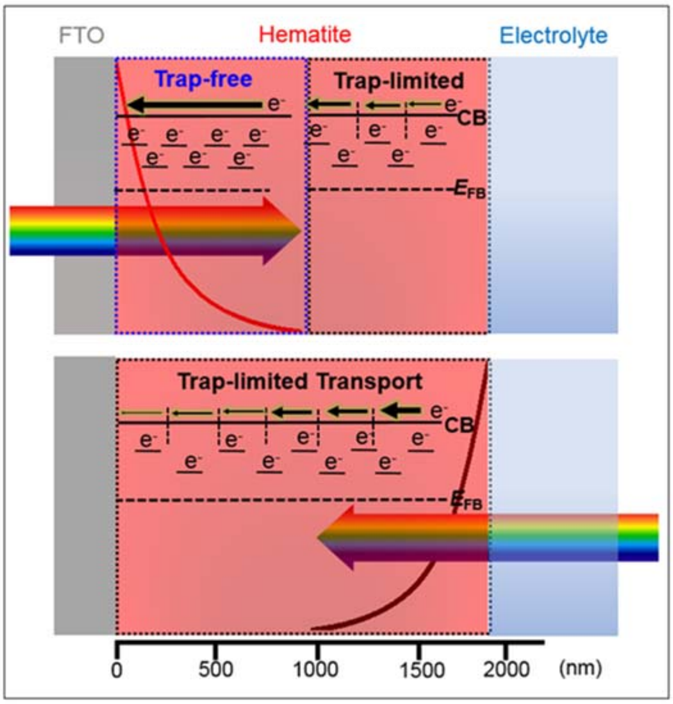

Supplementary Fig. 27 The proposed diffusion processes of charge carriers under different illumination modes. E_{FB} is the flat-band potential. Under back illumination, as a large number of charges is generated near the FTO region (Region 1 in Fig. 5d), most of the traps will be filled by electrons, thus enabling a trap-free transport in this region. The charges that are generated in or diffuse to Region 2 in Fig. 5d must go across a number of unfilled trap states (trap-limited transport), thus lowering the charge collection efficiency. Therefore, the improved current (~14%) in the thicker film (1500 nm), as compared with the 900-nm film, infers that the charges generated in Region 1 can travel large distances along the intimately connected MC. As larger film thicknesses (>1500 nm) should have more unfilled traps near the SEI, significant charge recombination will occur, resulting in the decrease of current density. Under front illumination, because most of the charges are generated in the region far away from the FTO side, the filled traps no longer exist near the FTO region. In such a case, all the generated charges travel across a larger number of unfilled traps via the trap-limited transport, thus leading to very poor current density due to significant recombination in thicker films (>900 nm).

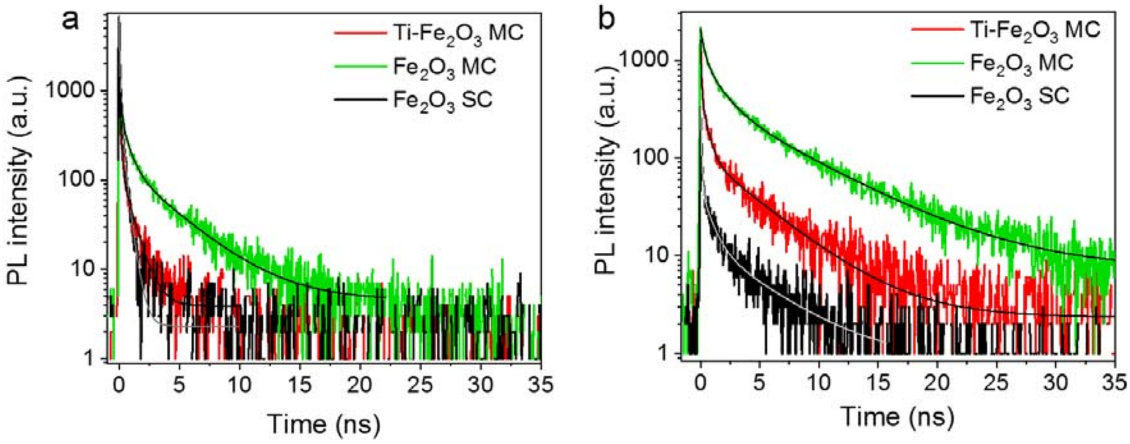

Supplementary Fig. 28 PL decay curves measured with longpass filter (>593 nm) (a) and bandpass filter (440–570 nm) (b).

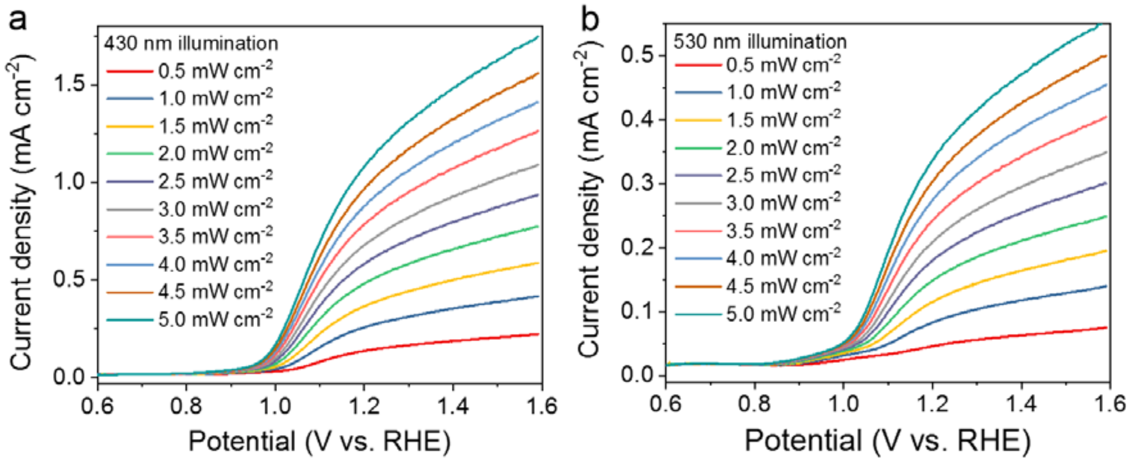

Supplementary Fig. 29 The current density-voltage curves of Ti-Fe_2O_3 MC photoanodes (with film thickness of 1.5 μm) under back illumination with monochromatic light ((a) 430 nm and (b) 530 nm) at different light intensities in 1.0 M NaOH.

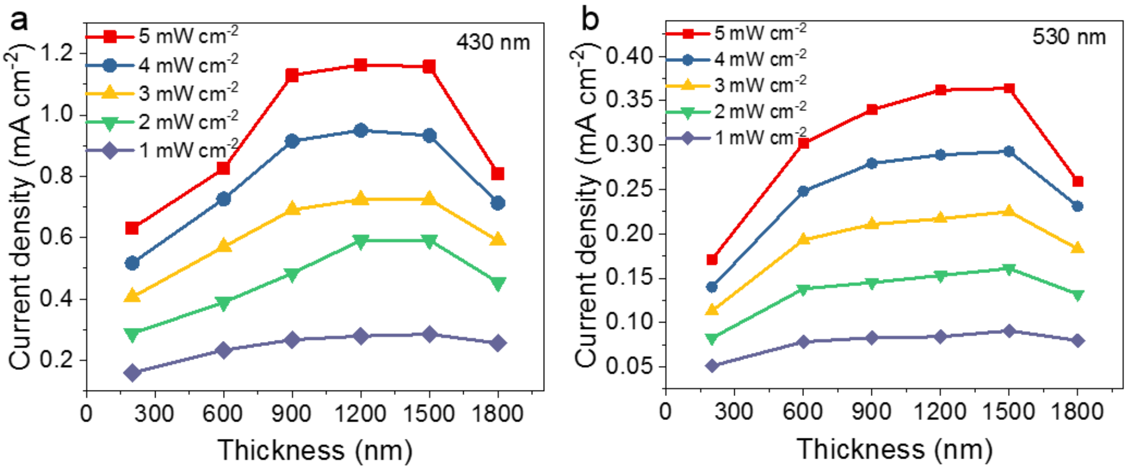

Supplementary Fig. 30 The thickness-dependent current densities under back illumination with monochromatic light ((a) 430 nm and (b) 530 nm) at different light intensities in 1.0 M NaOH.

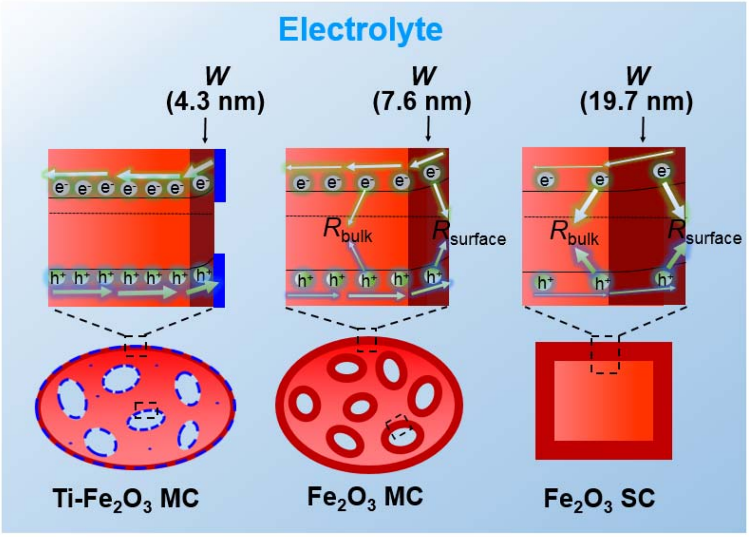

Supplementary Fig. 31 Illustration of the depletion regions at the semiconductor/electrolyte interfaces for Ti-Fe_2O_3 MC, Fe_2O_3 MC, and Fe_2O_3 SC.

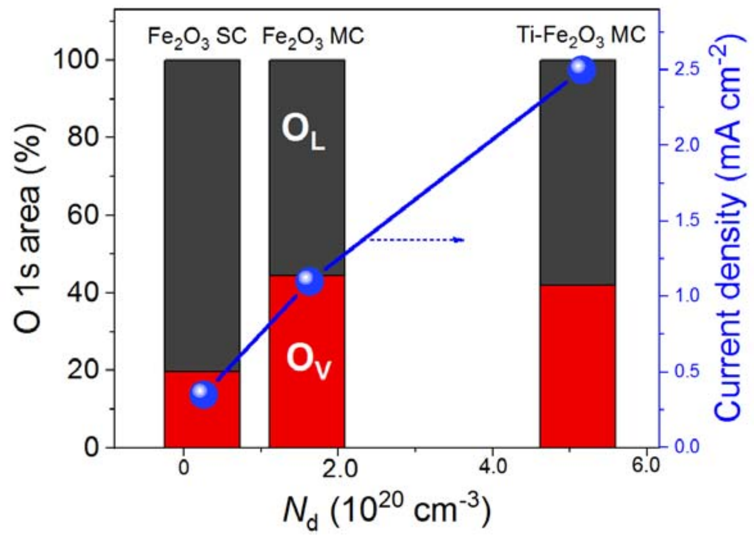

Supplementary Fig. 32 The correlation between carrier density (N_d) and surface V_O concentration estimated from the XPS O 1s as well as current density at 1.23 V vs. RHE for different photoanodes.

Supplementary Tables

Supplementary Table 1. Back Illumination Performance Compared to the Reported Hematite Photoanodes without Heterojunction and Additional Surface Treatment

**Photoanodes**	**Annealing temperature (°C)**	**Film thickness (nm)**	**Photocurrent density (mA cm^{-2}) (1.23 V vs. RHE)**	**Ref.**
Sn-Fe_2O_3 nanowire	800	370	0.45	19
Fe_2O_3/TiO_x nanorod	650	700	0.8	20
Sn-Fe_2O_3 nanowire	800	2500	0.6	21
Fe_2O_3 columnar grain structure	800	50	~0.59	22
Sn-Fe_2O_3	800	300	0.75	23
Ti-doped hematite nanorod	700	< 400	1.3	24
Fe_2O_3 rod crystal	800	275-400	0.32	25
Cd-Fe_2O_3 porous structure	700	1300-1800	~1.3	26
Ti-Fe_2O_3 nanowire	700	N/A	0.42	27
α-Fe_2O_3 thin film	600	175	0.36	28
α-Fe_2O_3 nanorod	700	650	0.202	29
nanoporous columnar hematite	N/A	270	0.75	30
Hydrothermal hematite nanorod	N/A	350	0.53	31
In^{3+}-treated α-Fe_2O_3 nanosheet	500	unknown	0.65	32
Ti-doped α-Fe_2O_3 nanosheet array	750	unknown	0.77	33
Mesoporous hematite thin film	820	930	1.1	34
Ti-Fe_2O_3 MC	700	1500	2.5	This work

Supplementary Table 2. Parameters Determined from Electrochemical Measurements

**Photoanode**	$\boldsymbol{R_s}$ **(Ω cm^{-2})**	$\boldsymbol{R_{ct}}$ **(Ω cm^{-2})**	$\boldsymbol{R_{trap}}$ **(Ω cm^{-2})**	$\boldsymbol{C_{bulk}}$ **(F cm^{-2})**	$\boldsymbol{C_{trap}}$ **(F cm^{-2})**	$\boldsymbol{N_d}$ **(cm^{-3})**	**Flat-band potential (V vs. RHE)**
Fe_2O_3 SC	100	2747	3345	8.15×10^{-6}	3.91×10^{-5}	2.4×10^{19}	0.24
Fe_2O_3 MC	98	576	2408	8.98×10^{-6}	7.16×10^{-6}	1.6×10^{20}	0.26
Ti-Fe_2O_3 MC	43	367	476	7.94×10^{-6}	7.90×10^{-6}	5.1×10^{20}	0.27

Supplementary Table 3. PL Properties Reported in the Literature

Sample	Excitation wavelength (nm)	Emission band (nm)	Peak wavelength (nm)	Assignment	Ref.
undoped film	520	580-650	610	band gap emission	35
nanorods	450	600-850	670	band-edge transition, red-shifted by 80 nm	36
$NiMnO_x$-loaded thin film	514	Broad	540	recombination of photoexcited holes with electrons occupying the singly ionized oxygen vacancies in α-Fe_2O_3	37
nanoblades	532	550-900	670	the surface effect	38
nanorods	450	600-800	670	band-edge transition (80 nm red shift)	39
Sn-doped film	360	N/A	468	unknown	40
nanoparticles	460	N/A	688	band edge emission	41
nanoflowers	410	N/A	590	band edge emission	42
mesocrystals	390	N/A	570	recombination of electrons and holes	43
nanowires	unknown	N/A	540/590/730	unknown (similar peak was observed, but was not explained)	44
mesoporous particles	unknown	548-550	N/A	electron-hole recombination	45
nanoparticles	488	500-700	530	self-trapped state	46
nanoparticles (5 nm in size)	480	500-750	577	quantum confinement and dielectric confinement for local optical transitions	47
nanorods	unknown	N/A	550	surface trapping states	48
nanoparticles (with average dimensions of 1 × 5 nm)	300/390	350-650	390	LMCT transitions	49

Supplementary Table 4. Kinetic Parameters for the Emission Decay[a]

Sample	a_1	τ_1 (ns)	a_2	τ_2 (ns)	a_3	τ_3 (ns)	$<\tau_{PL}>^a$ (ns)
Fe_2O_3 SC (without filter)	6215	<0.1	2914	0.2	154	1.7	0.4
Fe_2O_3 SC (with bandpass filter)	1210	<0.1	35.6	0.8	34	1.8	0.7
Fe_2O_3 SC (with longpass filter)	3750	<0.1	1418	0.2	1295	0.4	0.3
Fe_2O_3 MC (without filter)	2015	0.3	1383	1.3	506	6.4	4.1
Fe_2O_3 MC (with bandpass filter)	1006	0.3	706	1.6	353	7.0	4.9
Fe_2O_3 MC (with longpass filter)	1244	<0.1	489	0.5	143	3.2	1.9
Ti-Fe_2O_3 MC (without filter)	3365	<0.1	1044	0.5	207	3.6	1.9
Ti-Fe_2O_3 MC (with bandpass filter)	1105	<0.1	379	0.5	110	4.1	2.8
Ti-Fe_2O_3 MC (with longpass filter)	1114	<0.1	581	0.1	148	0.9	0.5

[a] The multiexponential decay curves were fitted using a nonlinear least-squares method with a three-component decay law described by $I(t) = a_1\exp(-t/\tau_1) + a_2\exp(-t/\tau_2) + a_3\exp(-t/\tau_3)$. The average lifetime ($<\tau>$) was than evaluated using the equation:

$$<\tau> = \sum_{i=1}^{i=n} a_i \tau_i^2 \, / \sum_{i=1}^{i=n} a_i \tau_i$$

Supplementary Note

All the applied voltage has been converted into the potential vs. RHE via the Nernst equation:

$$E_{RHE} = E_{Ag/AgCl} + 0.059 \times pH + E^{0}_{Ag/AgCl} \quad (1)$$

E_{RHE} represents the converted potential vs. RHE, and $E_{Ag/AgCl}$ is the applied potential vs. Ag/AgCl, $E^{0}_{Ag/AgCl}$ is 0.189 V at ambient temperature (25 °C), pH of the electrolyte is 13.6.

Incident photon to current efficiency (IPCE) can be calculated via the following equation:

$$\text{IPCE (\%)} = [1240 \times I(\lambda)/ \lambda \times J_{light}(\lambda)] \times 100\% \quad (2)$$

Where $I(\lambda)$ refers to the photocurrent density (mA cm^{-2}) at wavelength of λ, $J_{light}(\lambda)$ is the irradiance intensity (mW cm^{-2}) at wavelength (λ) of light.

The carrier density (N_d) can be calculated according to the following equation:

$$N_d = (2/e_0\varepsilon\varepsilon_0)[d(1/C^2)/dV]^{-1} \quad (3)$$

where e_0 is the electron charge, ε is the dielectric constant of hematite and ε_0 is the permittivity of vacuum, $d(1/C^2)/dV$ is the slop of the obtained Mott-Schottky curve.

The density of surface state (N_{SS}) can be calculated according to the following equation:

$$N_{SS}(E) = C_{trap}(E)/q \quad (4)$$

Supplementary Discussion

The correlation between N_d and V_O concentration on surface estimated from the XPS O 2p (Supplementary Fig. 19), as well as current density at 1.23 V vs. RHE (Fig. 3a), is summarized in Supplementary Fig. 32. It is clear that both the N_d and current density were highly increased by V_O formation and Ti modification. In general, the creation of V_O in metal oxides requires an external low oxygen environment. For example, Wang et al. recently reported that Ti-doped hematite photoanode with ~50% of V_O on the surface and N_d at 10^{18} cm^{-3} level can be created by annealing under N_2 environment.[15] For comparison, our Ti-Fe_2O_3 MC photoanode obtained by annealing in air can only create ~40% of V_O on the surface but reach a much higher N_d at 10^{20} cm^{-3} level (Supplementary Fig. 32), which would originate from the abundant V_O formed at the sintered interfaces inside the MC as proved by STEM-EELS (Fig. 2). They can serve as electron donor centers in the photoanode and thus suppress the bulk recombination effectively. Despite most of Ti species are deposited on the surface as TiO_2, ~2% (or less) of Ti^{4+} was estimated to be still located in the bulk of hematite, thus leading to ~3 times increase of N_d in Ti-Fe_2O_3 MC electrode as compared to Fe_2O_3 MC electrode. The TiO_2 layer (<1–4 nm) formed via thermal growth shows the same crystal orientation as the bulk hematite, which might contribute to the surface passivation or post-treatment for efficient charge transfer (Fig. 7a). It was reported that surface modification by atomic layer deposition of thin TiO_2 overlayer (1–2 nm thickness) on hematite can also effectively passivate the surface states and suppress the surface charge recombination.[50] Finally, we mentioned that there is no emission from rutile TiO_2, which is known to exhibit defect-mediated emission in the near-infrared region (~830 nm),[51] for Ti-Fe_2O_3 MCs.

According to the recent works using so-called 4D electron microscopy and transient absorption spectroscopy, long-lived O^- holes can be generated when hematite is excited with shorter wavelength light (e.g., ~400 nm).[52] For the MC samples with abundant V_O defects, the remaining two electrons from the O^{2-} site will occupy available Fe 3d states of the neighboring Fe ions. This structural change may shorten the local Fe−O bonds[53] and lead to greater Fe d and O p orbital hybridization, which corresponds to the chemical shifts verified by the XPS analysis (Supplementary Fig. 9c and 19). Therefore, the ligand-to-metal charge transfer (LMCT) transition (O 2p → Fe 3d) centered at 3.2 eV[54] can effectively occur when being excited by short-wavelength light. A recent study employing time-resolved microwave conductivity suggested that the 355 nm laser excitation of pure and metal-doped hematite films produces more mobile charges than excitation with 532 nm light that is not well matched with the LMCT transition.[55]

Recently, the excitation-wavelength-dependent lifetime of the photoexcited electrons was explained in terms of polaron-hopping theory.[56] The higher excitation energy provides more excess energy to the lattice, i.e., fewer polarons being formed by the non-thermal phonon bath, and thus the hopping rate of the polarons in equilibrium with mobile carriers will increase, resulting in the increased hopping radius and lifetime of carriers. However, this model cannot solely explain our finding that the nanosecond PL lifetimes were observed only for the MC samples.

Supplementary References

1. Duan, X., Mei, L., Ma, J., Li, Q., Wang, T., & Zheng, W. Facet-induced formation of hematite mesocrystals with improved lithium storage properties. *Chem. Commun.* **48**, 12204–12206 (2012).
2. Wang, H. et al. Photocatalytic properties dependent on the interfacial defects of intergrains within TiO_2 mesocrystals. *Chem. Eur. J.* **24**, 17105–17116 (2018).
3. Fujii, T. et al. In situ XPS analysis of various iron oxide films grown by NO_2-assisted molecular-beam epitaxy. *Phys. Rev. B* **59**, 3195–3202 (1999).
4. Lindsley, D. H. Experimental studies of oxide minerals. *Rev. Mineral Geochem.* **25**, 69–106 (1991).
5. Sanson, A. et al. Local vibrational dynamics of hematite (α-Fe_2O_3) studied by extended x-ray absorption fine structure and molecular dynamics. *J. Chem. Phys.* **140**, 224504 (2014).
6. Bocher, L. et al. Direct evidence of Fe^{2+}-Fe^{3+} charge ordering in the ferrimagnetic hematite-ilmenite $Fe_{1.35}Ti_{0.65}O_{3-\delta}$ thin films. *Phys. Rev. Lett.* **111**, 167202 (2013).
7. Muto, S. & Shiga, M. Application of machine learning techniques to electron microscopic/spectroscopic image data analysis. *Microscopy* **69** (2020) in press. DOI: 10.1093/jmicro/dfz036.
8. van Aken, P. A. & Lauterbach, S. Strong magnetic linear dichroism in Fe L_{23} and O K electron energy-loss near-edge spectra of antiferromagnetic hematite α-Fe_2O_3. *Phys. Chem. Miner.* **30**, 469–477 (2003).
9. Chen, S.-Y. et al. Electron energy loss spectroscopy and ab initio investigation of iron oxide nanomaterials grown by a hydrothermal process. *Phys. Rev. B* **79**, 104103 (2009).
10. Dennenwaldt, T. et al. Insights into the structural, electronic, and magnetic properties of $Fe_{2-x}Ti_xO_3/Fe_2O_3$ thin films with x = 0.44 grown on Al_2O_3 (0001). *J. Mater. Sci.* **50**, 122–137 (2014).
11. Gloter, A. et al. Electronic structure of titania-based nanotubes investigated by EELS spectroscopy. *Phys. Rev. B* **80**, 035413 (2009).
12. Dorian Hanaor, A. H. & Sorrell, C. C. Review of the anatase to rutile phase transformation. *J. Mater. Sci.* **46**, 855–874 (2011).
13. McCafferty, E., & Wightman, J. P. Determination of the concentration of surface hydroxyl groups on metal oxide films by a quantitative XPS method. *Surf. Interface Anal.* **26**, 549–564 (1998).
14. Wang, Z. et al. Understanding the roles of oxygen vacancy in hematite based photoelectrochemical process. *Angew. Chem. Int. Ed.* **58**, 1030–1034 (2019).
15. Zhou, Z. et al. Control of charge carriers trapping and relaxation in hematite by oxygen vacancy charge: Ab initio non-adiabatic molecular dynamics. *J. Am. Chem. Soc.* **139**, 6707–6717 (2017).
16. Odedairo, T. et al. Hexagonal sphericon hematite with high performance for water oxidation. *Adv. Mater.* **29**, 1703792 (2017).
17. Marusak, L.A., Messier, R., White, W. B. Optical absorption spectrum of hematite, α-Fe_2O_3 near IR to UV. *J. Phys. Chem. Solids* **41**, 981–984 (1980).

18. Ovcharenko, R., Voloshina, E., & Sauer, J. Water adsorption and O-defect formation on Fe_2O_3(0001) surfaces. *Phys. Chem. Chem. Phys.* **18**, 25560–25568 (2016).
19. Li, M. et al. Morphology and doping engineering of Sn-doped hematite nanowire photoanodes. *Nano Lett.* **17**, 2490–2495 (2017).
20. Bassi, P. S. et al. Understanding charge transport in non-doped pristine and surface passivated hematite (Fe_2O_3) nanorods under front and back illumination in the context of light induced water splitting. *Phys. Chem. Chem. Phys.* **18**, 3070–3078 (2016).
21. Qin, D.-D. et al. Facile solvothermal method for fabrication arrays of vertically oriented α-Fe_2O_3 nanowires and their application in photoelectrochemical water oxidation. *Energy Fuels* **25**, 5257–5263 (2011).
22. Jia, L. et al. α-Fe_2O_3 films for photoelectrochemical water oxidation – insights of key performance parameters. *J. Mater. Chem. A* **2**, 20196–20202 (2014).
23. Emin, S. et al. Photoelectrochemical water splitting with porous α-Fe_2O_3 thin films prepared from Fe-Fe-oxide nanoparticles. *Appl. Catal. A* **523**, 130–138 (2016).
24. Zhao X. et al. The influence of Ti doping on morphology and photoelectrochemical properties of hematite grown from aqueous solution for water splitting. *Energy Tech.* **6**, 2188–2199 (2018).
25. Walsh, D. et al. Simultaneous formation of FeO_x electrocatalyst coating within hematite photoanodes for solar water splitting. *ACS Appl. Energy Mater.* **2**, 2043–2052 (2019).
26. Bak, A. et al. Enhancing the photoelectrochemical performance of hematite (α-Fe_2O_3) electrodes by cadmium incorporation. *Appl. Catal. B* **110**, 207–215 (2011).
27. Franking, R. et al. Facile post-growth doping of nanostructured hematite photoanodes for enhanced photoelectrochemical water oxidation. *Energy Environ. Sci.* **6**, 500–512 (2013).
28. Eftekharinia, B. et al. Optimization of charge transport in a Co-Pi modified hematite thin film produced by scalable electron beam evaporation for photoelectrochemical water oxidation. *J. Mater. Chem. A* **5**, 3412–3424 (2017).
29. Li, L. et al. The effect of annealing regime and electrodeposition time on morphology and photoelectrochemical performance of hematite converted from nanosheet r-FeOOH. *J. Photochem. Photobio. A: Chem.* **369**, 8–15 (2019).
30. Eftekharinia, B. et al. Efficient nanoporous hematite photoanodes prepared by electron beam evaporation and Au modification. *ChemCatChem* **10**, 4665–4675 (2018).
31. Li, L. et al. The effect of fast and slow surface states on photoelectrochemical performance of hematite photoanodes fabricated by electrodeposition and hydrothermal methods. *J. Mater. Sci.* **54**, 659–670 (2019).
32. Bu, X. et al. Foreign In^{3+} treatment improving the photoelectrochemical performance of a hematite nanosheet array for water splitting. *Nanoscale* **9**, 17513–17523 (2017).
33. Ji, M. et al. Controlled growth of ferrihydrite branched nanosheet arrays and their transformation to

hematite nanosheet arrays for photoelectrochemical water splitting. *ACS Appl. Mater. Interfaces* **8**, 3651–3660 (2016).

34. Gonçalves R. H. et al. Magnetite colloidal nanocrystals: A facile pathway to prepare mesoporous hematite thin films for photoelectrochemical water splitting. *J. Am. Chem. Soc.* **133**, 6012–6019 (2011).
35. Zhang, M. et al. A facile strategy to passivate surface states on the undoped hematite photoanode for water splitting. *Electrochem. Commun.* **23**, 41–43(2012).
36. Shen, S. et al. Surface tuning for promoted charge transfer in hematite nanorod arrays as water-splitting photoanodes. *Nano Res.* **5**, 327–336 (2012).
37. Bhandary, N. et al. Enhanced photoelectrochemical performance of electrodeposited hematite films decorated with nanostructured $NiMnO_x$. *RSC Adv.* **6**, 35239–35247 (2016).
38. Yuan, L. et al. Morphological transformation of hematite nanostructures during oxidation of iron. *Nanoscale* **5**, 7581–7588 (2013).
39. Shen, S. et al. Effect of Cr doping on the photoelectrochemical performance of hematite nanorod photoanodes. *Nano Energy* **1**, 732–741 (2012).
40. Qin, D.-D. et al. Sn-doped hematite films as photoanodes for efficient photoelectrochemical water oxidation. *J. Mater. Chem. A* **3**, 6751–6755 (2015).
41. Alex, R. et al. Synthesis of biogenic hematite (a-Fe_2O_3) nanoparticles for antibacterial and nanofluid application. *RSC Adv.* **6**, 94206 (2016).
42. Wang, L. et al. Plasmon-induced hole-depletion layer on hematite nanoflake photoanodes for highly efficient solar water splitting. *Nano Energy* **35**, 171–178 (2017).
43. Chen, X. et al. Ag nanoparticles/hematite mesocrystals superstructure composite: a facile synthesis and enhanced heterogeneous photon-fenton activity. *Catal. Sci. Technol.* **6**, 4184–4191 (2016).
44. Li, Y. et al. Facile fire treatment of nanostructured hematite with an enhanced photoelectrochemical water splitting performance. *J. Mater. Chem. A* **4**, 14974–14977 (2016).
45. Demirci, S. et al. Fabrication and characterization of novel iodine doped hollow and mesoporous hematite (Fe_2O_3) particles derived from sol-gel method and their photocatalytic performances. *J. Hazardous Matel.* **345**, 27–37 (2018).
46. Zou, B. et al. Anomalous optical properties and electron-phonon coupling enhancement in Fe_2O_3 nanoparticles coated with a layer of stearates. *J. Phys. Chem. Solids* **58**, 1315–1320 (1997).
47. Zou, B. S. & Volkov, V. Surface modification on time-resolved fluorescences of Fe_2O_3 nanocrystals. *J. Phys. Chem. Solids* **61**, 757–764 (2000).
48. Li, F. et al. Construction of an efficient hole migration pathway on hematite for efficient photoelectrochemical water oxidation. *J. Mater. Chem. A* **6**, 23478–23479 (2018).
49. Li, F. et al. Ultrafast studies of photoexcited electron dynamics in γ- and α-Fe_2O_3 semiconductor nanoparticles. *J. Phys. Chem. B* **102**, 770–776 (1998).

50. Yang, X. et al. Improving hematite-based photoelectrochemical water splitting with ultrathin TiO_2 by atomic layer deposition. *ACS Appl. Mater. Interfaces* **6**, 12005–12001 (2014).
51. Pallotti, D. K. et al. Photoluminescence mechanisms in anatase and rutile TiO_2. *J. Phys. Chem. C* **121**, 9011–9021 (2017).
52. Su, Z. et al. Ultrafast element and oxidation-state mapping of hematite by 4D electron microscopy. *J. Am. Chem. Soc.* **139**, 4916–4922 (2017).
53. Zhou, Z. et al. Control of charge carriers trapping and relaxation in hematite by oxygen vacancy charge: *Ab initio* non-adiabatic molecular dynamics. *J. Am. Chem. Soc.* **139**, 6707–6717 (2017).
54. Hayes, D. et al. Electronic and nuclear contributions to time-resolved optical and X-ray absorption spectra of hematite and insights into photoelectrochemical performance. *Energy. Environ. Sci.* **9**, 3754–3769 (2016).
55. Kay, A. et al. Effect of doping and excitation wavelength on charge carrier dynamics in hematite by time-resolved microwave and terahertz photoconductivity. *Adv. Funct. Mater.* 1901590 (2019).
56. Carneiro, L. M. et al. Excitation-wavelength-dependent small polaron trapping of photoexcited carriers in α-Fe_2O_3. *Nat. Mater.* **16**, 819–825 (2017).
